# CD4/CD8 ratio and cytokine levels of the BAL fluid in patients with bronchiectasis caused by sulfur mustard gas inhalation

**DOI:** 10.1186/1476-9255-4-2

**Published:** 2007-01-16

**Authors:** Ali Emad, Yasaman Emad

**Affiliations:** 1 Associate professor of Medicine, Section of Pulmonary Diseases, Shiraz University of Medical Sciences, PO Box: 71345-1674, Shiraz, Islamic Republic of Iran; 2 Master of Sciences, Shiraz University, PO Box: 71345-1674, Shiraz, Islamic Republic of Iran

## Abstract

**Objective:**

To analyze cytokine levels in BAL fluid of patients with bronchiectasis due to mustard gas inhalation.

**Patients:**

29 victims with mustard gas-induced bronchiectasis and 25 normal veterans as control group.

**Intervention:**

PFTs,, high-resolution CT scans of the chest, analyses of BAL fluids for five cytokines (IL-8, IL-1β, IL-6, TNF-α, IL-12) and analyses of BAL fluids for cellular and flow-cytometric analysis of the phenotype of bronchoalveolar cells were performed in all cases.

**Results:**

CD4 lymphocytes expressed as percentage or absolute number were significantly higher in patients with bronchiectasis than in controls (32.17 ± 16.00 vs 23.40 ± 6.97%, respectively; p = 0.01; and 3.31 ± 2.03 vs 1.88 ± 0.83 × 10^3 ^cells/ml, respectively; p = 0.001). The CD4/CD8 ratio was significantly higher in patients with bronchiectasis than in controls (3.08 ± 2.05 vs 1.68 ± 0.78; p = 0.002).

There were significant differences in cytokine (IL-8, IL-1β, IL-6, TNF-α, IL-12) levels of BAL fluid between patients with bronchiectasis and healthy controls.

A significant correlation was observed between the HRCT scores and both the percentage and the absolute number of CD4 lymphocytes in BAL fluid in patients with bronchiectasis (r = -0.49, p = 0.009; r = -0.50, p = 0.008; respectively). HRCT scores showed a significant correlation with CD4/CD8 ratios (r = 0.54, p = 0.004) too.

Of measured BAL cytokines, only IL-8 (r = -0.52, p = 0.005) and TNF-aα (r = 0.44, p = 0.01) showed significant correlations with the HRCT scores.

**Conclusion:**

The increased levels of cytokines CD4 lymphocytes in the BAL fluid suggest the possible causative mechanism in the lung in sulfur mustard gas-induced bronchiectasis by the recruitment of neutrophils into the lung.

## Background

The toxicity of the chemical warfare blistering agent sulfur mustard (2,2'-dichlorodiethyl sulfide; SM) has been investigated for nearly a century [1,2]. This toxic gas can damage the eyes and respiratory tract when present in high doses [3].

Bronchiectasis, a chronic supportive lung disease characterized by irreversible dilatation of the bronchi and persistent suppurative sputum production, is a well-known late complication of sulfur mustard gas exposure in human [4]. Although the respiratory tract lesions represent the major debility after sulfur mustard exposure, only a few studies have investigated the pathophysiology of sulfur mustard-induced respiratory diseases, in particular the mechanisms involved in inflammatory processes.

T-lymphocytes have an essential role in many types of inflammatory response. It could, therefore, be anticipated that the lymphocytes may also play a role in the response to inhaled mustard gas. We undertook this study to investigate the role of T cell and proinflammatory cytokines in patients with bronchiectasis due to sulfur mustard gas inhalation. This study was designed to analyze bronchoalveolar lavage lymphocyte subsets and to determine the ratio of CD4 to CD8 lymphocytes in BAL fluid in these veterans with bronchiectasis. The levels of cytokines in patients having bronchiectasis were further studied to correlate with the disease severity expressed as HRCT scores.

## Methods

### Patient population

Of all the veterans admitted to our university teaching hospital in 1986 with a single heavy exposure to sulfur mustard gas, 29 male patients with bronchiectasis were enrolled into this study. The patients' exposure to sulfur mustard gas had been confirmed by studies on their urine and vesicular fluid in 1986. The etiology of bronchiectasis was post-sulfur-mustard gas inhalation. Patients were characterized as having bronchiectasis based on a history consistent with the disease and a computed tomographic (CT) scan of the chest showing pathological changes consistent with bronchiectasis [5]. All patients had a persistent cough and daily sputum production. All of the patients were lifetime nonsmokers.

All cases had bilateral bronchiectasis. Patients were excluded from the study if they had suffered an exacerbation of their symptoms during the preceding 4 weeks. None of the 29 patients had received antibiotic therapy or oral steroids within the four week period preceding the study.

None of the patients had any evidence of connective tissue disorders, sarcoidosis, eosinophilic granuloma, pneumoconiosis, carcinomatosis, or lymphoma. They signed a written informed consent form and underwent a thorough history and physical examination. A chest roentgenogram, an ECG, and a high-resolution CT (HRCT) of the chest were obtained in each patient. Approval was obtained from the local ethics committee. This study was done in 2005.

### Measurement of pulmonary function tests (PFTs)

Pulmonary function tests were performed. These tests were measured through spirometric assessment according to the standards advocated by the American Thoracic Society [6]. Prior to bronchoscopy, an experienced physician did all spirometric measurements (FUDAC 50; Fukunda Sangyo; Chiba, Japan) in all patients and subjects. Each patient was well trained to give his best effort. After 15 min of resting, three spirometric measurements were done at 1-min intervals; the highest values measured were reported. Results were expressed as percentage predicted based on accepted reference standards [7,8].

### High-resolution computed tomographic examination

The HRCT scans were done with 1- to 1.5-mm sections taken at 1-cm intervals through the entire thorax and were reconstructed using a bone algorithm. The HRCT scans of the patients was assessed and scored by the same consultant pulmonary radiologist, who was blinded to all other details concerning the patient. Each lobe of both lungs was graded for bronchiectasis changes on a 0–3 scale (the lingula was scored as a separate lobe), giving a maximum of 18 points: 0: no bronchiectasis; 1: one or no bronchopulmonary segment involved; 2: more than one bronchopulmonary segment involved; and 3: gross cystic bronchiectasis [9].

### Healthy control subjects

The control group consisted of 25 normal, healthy nonsmoking veterans with a mean (± SD) age of 37.37 ± 4.56 years old (range, 29 to 48 years old); the control subjects had participated in the Iran-Iraq War but had not been exposed to mustard gas. All subjects voluntarily entered the study and signed an informed consent form before their enrollment. All subjects had a complete history and physical examination. No subjects had a history of exposure to organic or inorganic dusts. In addition to obtaining a chest radiograph, HRCT scan and an ECG for each subject, PFTs were measured prior to bronchoscopy as well. Asymptomatic healthy control subjects had no evidence of chronic disease or airflow obstruction and spirometric test results were within normal limits. The examination of BAL cells and the determination of T cells and cytokine levels were carried out for all cases using the same techniques as described for the patients group.

### Bronchoscopy and bronchoalveolar lavage (BAL)

All of the patients underwent a bronchoscopic examination. Before any lung biopsy specimens were taken, BAL was performed using a flexible fiberoptic bronchoscope (Olympus BF1T; Tokyo, Japan). The lavages were collected this year (20 years after exposure). Each patient was premedicated with atropine (0.75 mg IM) before the procedure. Mild sedation was achieved with intravenously administered midazolam, and supplementary oxygen was given throughout the procedure. Patient oxygenation was monitored by pulse oximetry. After applying 4% lignocaine spray to the nose and throat of the patient, the flexible bronchoscope was introduced. Local anesthesia of the larynx was achieved with topical 4% lignocaine, whilst a 2% solution was used below the vocal cords to suppress coughing. The bronchoscope was wedged for lavage in the middle lobe segmental bronchus, and four 60-ml aliquots of sterile physiologic saline solution warmed to 37°C were infused. The fluid was immediately recovered by gentle suction after each instillation. The first aliquot reflecting a bronchial sample was discarded, while the others were pooled for our study. One milliliter of recovered lavage fluid was processed for bacterial and fungal culture by routine methods. Then, the BAL fluid was passed through monolayer surgical gauze to eliminate mucus. One small aliquot of this fluid was utilized to count the total cell number, and another aliquot was spun in a cytometer at 400 revolutions/min for 10 min. The cell pellet was washed once in Hanks' balanced salt solution (without calcium and magnesium). A May-Grünwald-Giemsa stain smear was used to identify the differential profiles after cytospin preparation. Total cell counts were determined with a hemocytometer. The differential cell count of lymphocytes, neutrophils, macrophages, and eosinophils was made under light microscopy × 1,000 by counting approximately 300 cells in a random field. The result was expressed as cells × 10^3^/ml. The unconcentrated supernatant was frozen at -70°C before the protein was measured.

### Flow-cytometric analysis of the phenotype of bronchoalveolar cells

In order to identify the proportions of T lymphocytes, CD4, CD8 T cells, B cells and natural killer (NK) cells subpopulations of BAL fluid, cells were simultaneously stained with fluorescein isothiocyanate or (FITC) and phycoerythrin-conjugated (PE) monoclonal antibodies (anti-IgG1, -IgG2a, -CD3, -CD4, -CD8, -CD19, -CD56) (Beckon Dickinson, Mountain View, CA) according to the manufacturer's protocol. The relative ratio of CD4 or CD8 in CD3-positive cells was assayed by a dual-color analysis. Data were obtained and analyzed using Becton Dickinson BD LYSYS II and Cytometric Bead Array (CBA) software (San Jose, CA).

#### Measurement of the BAL cytokines

The levels of cytokines in BAL fluid were assayed using Becton Dickinson (BD) Cytometric Bead Array™ (CBA; BD Biosciences, San Jose, CA) [10] according to manufacturer's instructions with an antibody (PharMoingen, San Diego, CA) against one of the five cytokines (Human Inflammation Kit: IL-8, IL-1β, IL-6, TNF-α, IL-12, BD Biosciences). The lower limits of detection of the kit (supplier's data) were as follows: TNF-α, 0.085 pg/ml; IL-1β, 0.083 pg/ml; IL-6, 0.080 pg/ml; IL-8, 3.0 pg/ml, and IL-12, 1.9 pg/ml.

### Statistical analyses

Data are presented as mean ± SD unless otherwise stated. Comparisons were made using the Mann-Whitney nonparametric test for continuous data and by x^2 ^analysis for discontinuous data. Correlations between different parameters were determined by Spearman's rank correlation coefficient. P < 0.05 was considered significant.

## Results

### Demographics

The characteristics of the population are summarized in Table 1. The age of the two groups did not significantly differ (p = 0.37). HRCT scores showed a mean bronchial dilatation (± SD) score of 9.37 ± 147 (range, 6 to 11). No control subject had an obstructive or restrictive pattern in his PFTs.

**Table 1:** Characteristics of the 29 bronchiectasis patients and 25 healthy subjects enrolled in the study*

	Controls (no = 25)	Bronchiectasis (no = 29)	p Value
Age (years), mean ± SD	36.24 ± 4.78	37.37 ± 4.56	0.37
HRCT score*, mean ± SD	0.00 ± 0.00	9.37 ± 147	< 0.0001
HRCT score*, range	0 ± 0	6 ± 11	-
FEV_1_, % of predicted	97.53 ± 4.76	71.82 ± 6.52	< 0.0001

* Values are mean ± SD.

### BAL fluid cellular constituents

Bacterial agents were classified into microorganisms with potential pathogenicity (MPP) or microorganisms with nonpotential pathogenicity, as described elsewhere (34). Only microorganisms with potential pathogenicity (MPP) with counts of ≥ 10^3 ^cfu × ml^-1 ^in BALF were regarded as significant. The pathogens recovered from BALF of patients with bronchiectasis were P. aeruginosa (no = 2), Haemophilus influenzae (no = 3), Serratia marcescens (no = 1), and S. aureus (no = 2). Culture results in the control group showed negative.

The cellular contents of BAL fluids in both groups are shown in Table 2. Patients with bronchiectasis had a higher total cell count than the normal group, but it was not significant (p = 0.31). When compared to healthy controls, total and differential cell counts in patients with bronchiectasis showed, an increase both of neutrophil percentage (p < 0.0001) and neutrophil absolute number (13.69 ± 3.26 versus 2.04 ± 1.06 × 10^3 ^cells/ml; p < 0.0001).

**Table 2:** Comparison of bronchoalveolar lavage fluid cellular constituents of bronchiectasis due to sulfur mustard gas inhalation versus the control group*

	Controls (n = 25)	Bronchiectasis (n = 29)	p Value
Recovery of BAL fluid, ml	160.48 ± 12.64	155.44 ± 17.28	0.23
Number of cells (× 10^3^)	88.96 ± 7.59	91.89 ± 12.65	0.31
Macrophages (× 10^3^)	78.00 ± 8.24	67.62 ± 11.79	0.0005
Lymphocytes (× 10^3^)	8.40 ± 3.50	10.00 ± 2.08	0.04
Neutrophils (× 10^3^)	2.04 ± 1.06	13.69 ± 3.26	< 0.0001
Eosinophils (× 10^3^)	0.52 ± 0.58	0.58 ± 0.68	0.70

* Values are mean ± SD.

On comparison of the airway inflammatory characteristics between patients with bronchiectasis colonized by MPP (n = 8) and non-colonized patients with bronchiectasis (n = 21), we observed that the group of patients with MPP in the airways had higher BAL neutrophil count and percentage, and a higher BALF concentration of IL-8., although the differences did not reach statistical significance (Table 3). We observed that patients with bronchiectasis and negative cultures for MPP in the BALF had a more intense inflammatory reaction than did control subjects, with a higher percentage of neutrophils and higher concentrations of IL-8 and other cytokine profiles in BALF (Table 3).

**Table 3:** Total differential cell and neutrophil counts and IL8 concentrations from the BAL fluid of patients with bronchiectasis with or without microorganisms with potential pathogenicity (MPP) *

	Control Subjects	Noncolonized Patients	Colonized Patients
Subjects, no	25	8	21
Number of cells (× 10^3^)	88.96 ± 7.59	91.86	92.00‡
Neutrophils (× 10^3^)	2.04 ± 1.06	14.54†	16.63‡
Neutrophils, %	2.33 ± 1.29	13.09†	15.25‡
IL-8 (pg/ml)	1.04 ± 1.17	35.86†	41.75‡

* Values are mean ± SD.

† p = 0.05 comparing control subjects versus noncolonized patients

‡ p = 0.05 comparing colonized and noncolonized patients

Lymphocyte absolute number showed a week significant increase in bronchiectasis patients when compared to healthy controls (p < 0.04). The absolute number of alveolar macrophages, however, was significantly lower in patients with bronchiectasis than in the control group (p < p = 0.0005, respectively).

### Lymphocyte subsets in BAL Fluid

The summarized results of the lymphocyte phenotyping in the BALF of both the control and patients with bronchiectasis are observed in Table 4.

**Table 4:** Lymphocyte subpopulations in bronchoalveolar lavage from patients with bronchiectasis due to mustard gas exposure and normal subjects*

	Controls (n=25)	Bronchiectasis (n=29)	p Value
CD3 cells (%)	46.39 ± 15.03	48.15 ± 13.34	0.65
CD3 cells (10^3 ^cells/ml)	3.72 ± 1.62	4.82 ± 1.81	0.02
CD4 cells (%)	23.40 ± 6.97	32.17 ± 16.00	0.01
CD4 cells (10^3 ^cells/ml)	1.88 ± 0.83	3.31 ± 2.03	0.001
CD8 cells (%)	15.76 ± 6.84	11.84 ± 4.41	0.01
CD8 cells (10^3 ^cells/ml)	1.16 ± 0.37	1.13 ± 0.35	0.82
CD4/CD8 (ratio)	1.68 ± 0.78	3.08 ± 2.05	0.002
B cells (%)	5.56 ± 6.76	3.58 ± 5.55	0.24
B cells (10^3 ^cells/ml)	0.52 ± 0.58	0.31 ± 0.47	0.15
NK cells (%)	2.83 ± 6.34	1.49 ± 3.86	0.34
NK cells (10^3 ^cells/ml)	0.20 ± 0.40	0.13 ± 0.35	0.55

* Values are mean ± SD.

In patients with bronchiectasis there were increased absolute numbers of CD3 T lymphocytes compared with normal controls (4.82 ± 1.81 versus 3.72 ± 1.62 × 10^3 ^cells/ml, respectively; p = 0.02), while the percentage of CD3 cells showed no significant difference between the two groups (48.15 ± 13.34 versus 46.39 ± 15.03; p = 0.65).

The profile of T-cell subtypes showed that when data were expressed as percentage or as absolute number of positive cells, CD4 lymphocytes were significantly higher in patients with bronchiectasis than in healthy controls (32.17 ± 16.00 versus 23.40 ± 6.97%, respectively; p = 0.01; and 3.31 ± 2.03 versus 1.88 ± 0.83 × 10^3 ^cells/ml, respectively; p = 0.001). The proportion of CD8 cells in BAL fluid was significantly diminished in patients with bronchiectasis compared to the control group (11.84 ± 4.41 versus 15.76 ± 6.84; p = 0.01), while the absolute number of CD8 cells showed no significant difference between the two groups (1.13 ± 0.35 versus 1.16 ± 0.37; p = 0.82).

Consequently, the CD4/CD8 ratio was significantly higher in patients with bronchiectasis than in healthy controls (3.08 ± 2.05 versus 1.68 ± 0.78; p = 0.002).

When B and NK (neural killer) alveolar lymphocytes were evaluated either as a percentage or as absolute number, no significant differences were found between the patients with bronchiectasis and the control group (See Table 4).

### Comparison of BAL fluid cellular constituents with HRCT scores

**Figure 1:**
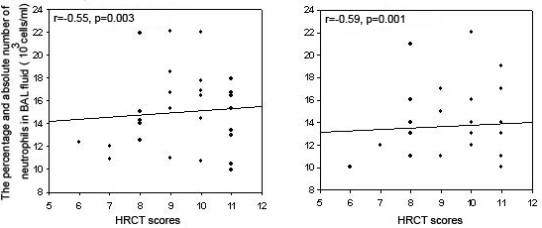
Relationship between the percentage and absolute number of neutrophils in BAL fluid and the HRCT scores in patients with bronchiectasis due to mustard gas inhalation. There were significant, positive correlations between the percentage (r = -0.55, p = 0.003) and the absolute number of neutrophils (r = -0.59, p = 0.001) in BAL fluid and HRCT scores.

A significant positive correlation was observed between HRCT scores and both the percentage and the absolute number of neutrophils in BAL fluid in patients with bronchiectasis (r = -0.55, p = 0.003; r = -0.59, p = 0.001; respectively) (Fig 1). No correlation was found between HRCT scores and the other BAL cells.

### Comparison of BAL Lymphocyte subpopulations with HRCT scores

**Figure 2:**
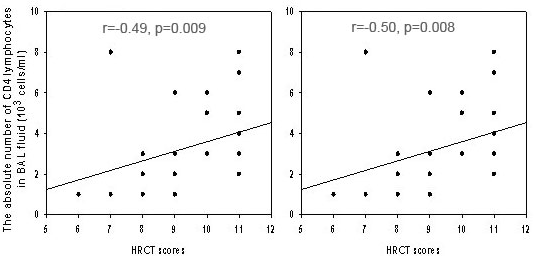
Relationship between the percentage and absolute number of CD4 lymphocytes in BAL fluid and the HRCT scores in patients with bronchiectasis due to mustard gas inhalation. There were significant, positive correlations between the percentage (r = -0.49, p = 0.009) of and the absolute number of CD4 lymphocytes (r = -0.50, p = 0.008) in BAL fluid and HRCT scores.

A significant positive correlation was observed between the HRCT scores and both the percentage and the absolute number of CD4 lymphocytes in BAL fluid in patients with bronchiectasis (r = -0.49, p = 0.009; r = -0.50, p = 0.008; respectively) (Fig 2). HRCT scores showed a significant correlation with CD4/CD8 ratios (r = 0.54, p = 0.004) too. No correlation was found between HRCT scores and the other BAL cells.

### Cytokine levels in BAL fluid samples

The BAL fluid levels of the cytokines (IL-8, IL-1β, IL-6, TNF-α, IL-12) were found to be significantly elevated in patients of bronchiectasis when compared with controls (p < 0.0001) (Table 5).

**Table 5:** Cytokine levels in bronchoalveolar lavage from normal subjects and patients with bronchiectasis*

	Controls (n=25)	Bronchiectasis (n=29)	p Value
IL-8 (pg/ml)	1.04 ± 1.47	37.48 ± 16.65	< 0.0001
IL-6 (pg/ml)	0.003 ± 0.01	2.53 ± 0.93	< 0.0001
IL-12 (pg/ml)	0.00 ± 0.00	1.90 ± 0.51	< 0.0001
IL-1β (pg/ml)	0.00 ± 0.00	3.71 ± 1.27	< 0.0001
TNF-α (pg/ml)	0.003 ± 0.01	1.30 ± 0.38	< 0.0001

* Values are mean ± SE.

### Comparison of HRCT scores and cytokine levels in BAL fluid

**Figure 3:**
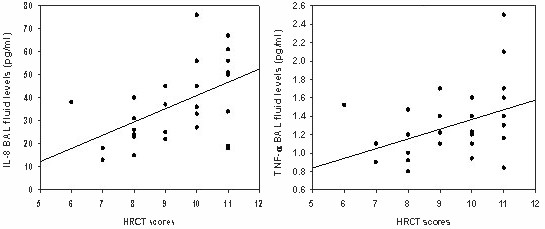
Relationship between the IL-8 and TNF-α levels in BAL fluid and the HRCT scores in patients with bronchiectasis caused by mustard gas inhalation. There were significant, positive correlations between the IL-8 (r = -0.52, p = 0.005) and TNF-α (r = -0.44, p = 0.01) in BAL fluid and HRCT scores.

Of measured BAL cytokines, only IL-8 (r = -0.52, p = 0.005) and TNF-α (r = 0.44, p = 0.01) showed significant correlations with the HRCT scores (Fig 3).

### Association between airway inflammation and FEV1

There was no significant difference in FEV1 between those with or without detectable infection (p = 0.66). FEV1 (percentage predicted) correlated negatively with both the number and the percentage of neutrophils (r = 0.53, p = 0.004) and IL-8 levels in BAL fluid of patients with bronchiectasis (r = 0.39, p = 0.03). A significant negative correlation was also observed between the FEV1 (percentage predicted) and both the percentage and the absolute number of CD4 lymphocytes in BAL fluid in these patients (r = 0.50, p = 0.007; r = -0.41, p = 0.02; respectively). FEV1 (% predicted) showed a negative significant correlation with CD4/CD8 ratios too (r = 0.40, p = 0.03). There was no apparent correlation between the other different inflammatory parameters evaluated in the BAL fluid and the FEV1 (percentage predicted) in patients with bronchiectasis (data not shown).

## Discussion

Bronchiectasis is the major cause of disability and mortality in mustard victims [4,11]. The main aim of this study was to compare both levels of BAL lymphocyte subpopulations and cytokines with the HRCT scores in patients with bronchiectasis due to sulfur mustard gas inhalation. To the best of the authors' knowledge, this is the first study in which BAL Lymphocyte subpopulations and cytokine levels have been evaluated in these victims with bronchiectasis.

This study revealed that patients with bronchiectasis, when compared to healthy subjects, were characterized by an increase in neutrophils in BAL fluid (Table 1). A positive correlation between either the percentage or the absolute number of BAL fluid neutrophils and the HRCT scores from patients with bronchiectasis was demonstrated (Fig 1). These data also clearly demonstrate that the degree of lung function impairment closely related to neutrophilic alveolitis in patients with bronchiectasis. It might act as a triggering factor in the development of bronchiectasis in mustard gas-exposed patients. The large numbers of neutrophils present in airspaces of the lung in post-sulfur-mustard gas inhalation bronchiectasis may be likely sequestered in response to chronic bacterial infection (See Table 2 and Table 3). This study showed positive BAL fluid cultures in only 8 patients. Interestingly, our findings suggest that airway inflammation may occur even in the absence of colonization as demonstrated by the significant increase in levels of the different inflammatory mediators between patients with negative BAL cultures and control subjects (Table 3). This study may confirm the presence of airway neutrophilia, despite negative cultures for the usual bronchiectasis-associated pathogens, suggesting that inflammation may even precede infection (See Table 3). In other words, quantitative bacteriological cultures in this study may clarify the role of a previous mustard gas exposure. Among the different cell types involved in the airway inflammation, neutrophils are recognized to play a central role by releasing proinflammatory mediators, such as reactive oxygen species and proteolytic enzymes [37]. It is suggested that infection and inflammation become intimately linked in the course of the disease, with each exacerbating the other. Thus, a vicious circle of infection and inflammation is established that is mainly responsible for progressive and irreversible lung damage in our patients with bronchiectasis. The finding of this study (neutrophilic inflammation) is similar to those described for patients with cystic fibrosis (CF) in the literature [35]. The activated neutrophils are the primary effector cells for the pathogenesis of CF lung disease [36,37].

Analysis of T-lymphocyte subsets in BAL fluid indicates that, in patients with bronchiectasis due to mustard gas exposed, both the percentage and total numbers of CD4 T cells increase and the percentage of CD8 T cells decreases (See Table 4). This study on BAL fluid has also shown that the CD4/CD8 ratio is significantly increased in patients with bronchiectasis (Table 4).

CD8 or CD4 T cells infiltrate the lung in many clinical conditions. An increase of T lymphocytes in airways in patients with cystic fibrosis (CF) has been documented (36). It is likely that secondary to infiltration by CD8+ T cells in the respiratory tract of HIV-infected children with bronchiectasis, pattern of gene expression could be changed in respiratory epithelium, leading to parenchymal damage by a complex of infectious and inflammatory reactions [38]. Activation of CD8+ cells in the airway of patients with diffuse panbronchiolitis has been also documented [39]. A predominant CD4+ T cell infiltrate in the bronchial mucosa in bronchiectasis patients has been observed [40].

Mustards are very lipophilic and can therefore penetrate epithelial tissues easily. The cellular damage and death is thought to be mediated via the alkylation of DNA, which leads to DNA strand breaks and apoptosis [41]. Human cells exposed in culture to mustard gas show concentration dependent decreases in cell proliferation, DNA synthesis, protein synthesis and NAD^+ ^and ATP levels [42]. The clinical manifestations of mustard gas exposure result from multiple mechanisms, although the exact pathways are not yet elucidated [43]. The columnar cells of the epithelial lining of the upper respiratory tract in an acute heavy exposure may be damaged by this toxic warfare gas. The shedding of the columnar cells may be accompanied by peribronchial edema, hyperemia of the blood vessels, cellular infiltrations in the submucosa and serious vacuolization and disorganization of cytoplasma and nuclear structures [44]. These reactions may result in physiological, metabolic and genetic failure of cellular functions. As it has been shown in this study, these may cause an inflammatory infiltrate (mostly T-lymphocytes and neutrophils) in the airways. In addition to T cells, other inflammatory cell types such as neutrophils and macrophages are probably essential in the initial inflammatory process leading to the breakdown of lung tissue, perhaps producing peptides eventually recognized by T cells as antigenic. This would provide an explanation for the T-cell inflammation. Once activated, T cells are present in the lung, and their effector functions would include the attraction and enhancement of the inflammatory function in other inflammatory cells like neutrophils and macrophages. Additionally, activation of several proteases and proinflammatory cytokines is involved in sulphur mustard injury.

However, the stimuli and the mechanisms that mediate CD4 T-cell recruitment to the lung of our patients with bronchiectasis are not understood. How CD4 T cells, which are recruited to the lungs of these patients, are triggered to express their effector functions, and how these functions are downregulated, also is not known precisely. But it is possible that injury to the airways epithelium, which is the first structure encountered by sulfur mustard gas, promotes and perpetuates inflammation in the airways. It is likely that the alkylation of DNA and the shedding of epithelial cells promote an initial neutrophilic inflammation that by diverse mechanisms (eg, proteases or oxidation) damages lung cells. Airway injury may lead to structural alterations in self-antigens that create partially cross-reactive neoantigens and to the release of anatomically sequestered antigens that would be recognized by autoreactive T cells, thereby inducing their activation and proliferation [45,46]. It is possible that direct tissue damage from sulfur mustard gas inhalation, known to cause antigen release. It may be also hypothesized that in patients with sulfur mustard gas-induced bronchiectasis an excessive recruitment of CD4 T lymphocytes may occur in response to recurrent or persistent bacterial infections, and this excessive response may play a role for the development of lung damage. Regardless of how the inflammatory process is initiated and the temporal relationship between infection and inflammation in sulfur mustard gas-induced bronchiectasis, this study shows that persistent inflammatory response in the airways plays a central role in the progression of lung damage.

It should be emphasized that in this study, CD4+ T lymphocytes are the predominant lymphocyte cells involved (See Table 4), whereas in COPD, CD8+, T lymphocytes, and macrophages are predominantly involved [47]. Comparison of results of this study with those patients with sulfur mustard gas-induced pulmonary fibrosis reveals, that CD8 T cells in BAL fluid were surprisingly significantly elevated in patients with pulmonary fibrosis (The methods of both studies are similar to each other) [48]. The observed variability in terms of CD4 and CD8 T lymphocytes are possibly linked to a number of factors such as: the concentration of inhaled toxin, the site of deposition of the toxin in the respiratory system, presence of infections, and differences in patient's vulnerability and susceptibility to the toxin.

We measured the relationship between T-lymphocyte subsets and HRCT scores in the patients with bronchiectasis due to mustard gas inhalation. The percentage and the absolute number of CD4 T cells highly correlated with the percentage of HRCT scores (r = -0.49, p = 0.009; r = -0.50, p = 0.008; respectively) (See Fig 2). Therefore, we present a set of observations that reveal that patients with more advanced lung function impairment as HRCT pathological findings, tended to reveal higher CD4/CD8_ratios. Therefore, It is possible that increased CD4 T cells in the BAL fluid may contribute to parenchymal destruction and therefore to the development of repeated exacerbations in these patients by releasing cytokines capable of increasing the susceptibility of target cells to cytotoxicity, or by secreting chemokines that attract other cells to the site of inflammation [12,13]. The significant correlation observed in our veterans between increased number of CD4 T cells in the BAL fluid and the HRCT scores supports this hypothesis (See Fig 2). These findings support a role for cell mediated immune mechanisms in the pathogenesis of ongoing airways damage in sulfur mustard gas-induced bronchiectasis.

It has been showed that cytokines produced by bronchial, bronchiolar, alveolar epithelial cells, alveolar macrophages and neutrophils are involved in the inflammatory response [14,15]. The equilibrium between the proinflammatory cytokines tumour necrosis factor (TNF-α), IL-12, IL-8, IL-6 and anti-inflammatory cytokines such as IL-10 is essential for directing the immune response [16,17].

It is apparent from the present findings that the levels of the pro-inflammatory cytokines increased significantly in patients with bronchiectasis compared with healthy controls (p < 0.0001) (Table 5). A significant elevation in IL-8 was seen between these groups. Significantly elevated levels of proinflammatory IL-6, TNF-α, and IL-12 were likewise noted in cases with bronchiectasis versus healthy controls.

Recruitment of neutrophils may lead to an increased and persistent inflammatory state in the alveolar region. It is likely that neutrophil accumulation in the BAL fluid of patients with bronchiectasis is driven by increased release of cytokines exerting a chemotactic effect on these cells. This suggests a key role for these cytokines in neutrophil influx into lung tissue and sustaining the intense local inflammatory response in the affected bronchial tree [13].

Tumor necrosis factor-α (TNF-α) is a proinflammatory cytokine predominantly released by macrophages and monocytes but also other cells produce TNF-α such as neutrophils and lymphocytes [18-20]. A role for in amplifying the inflammation of COPD has been suggested [52]. TNF-α has a broad spectrum of inflammatory effects relevant to COPD, resulting in activation of neutrophils, monocytes, macrophages, epithelium, mucus secretion, and destruction of lung parenchyma through release of proteinases [52]. TNF-α is known to stimulate various cells, including alveolar macrophages, for increased IL-8 production [21]. These observations, together with our finding of the elevation of IL-8 in BAL fluid in sulfur mustard gas-induced bronchiectasis patients, suggest that IL-8 plays an important role in the cell accumulation seen in the lung in this disease (Table 5). TNF-α is also a chemotactic agent for neutrophils [22]. In the cytokine network model in the lung, alveolar cells initially respond to a stimulus by secreting TNF-α and/or IL-1β. These cytokines then act in an autocrine or paracrine fashion, leading to IL-6 and IL-8 release and triggering an inflammatory response. On the other hand, IL-6 has been shown that it inhibits apoptosis [23]. Therefore, inefficient clearance of apoptotic cells results in secondary necrosis of cells and exacerbation of the inflammatory response [24]. Several cytokines known to stimulate IL-6 release include IL-1, TNF-α [25].

The participation of IL-8 has been reported in various diseases and conditions [26-28]. Among the recruitment factors for neutrophils IL-8 is by far the most potent (29). Since IL-8 can activate neutrophils by increasing degranulation and neutrophil elastase release, our attention has been focused on its pivotal and fundamental responsibility for bronchial destruction in bronchiectasis due to sulfur mustard gas inhalation [30].

Significant increased BAL fluid IL-8 level in patients with bronchiectasis could be related to the neutrophilic inflammatory response and alveolitis observed in response to acute massive inhalation of sulfur mustard gas (Table 5). The significant correlations between the concentration of IL-8 and either both, absolute count of and the percentage of neutrophils in BALF (p = 0.03 and p = 0.04, respectively) (data not mentioned in result) and the HRCT scores may approve this relationship (See Fig 3).

IL-12 is a heterodimeric cytokine that plays a key role in determining the nature of immune response to exogenous or endogenous antigens [31]. It can activate inflammatory cells like T cells, natural killer cells and can induce cytokines such as interferon-γ, which in turn promotes IL-12 production and macrophage activation [31,32]. An overabundance of TNF-α or IL-12 is correlated with the development of the inflammatory activity and the tissue damage. In this study, the positive correlation between the TNF-α levels in BAL fluid and the HRCT scores in patients with bronchiectasis caused by mustard gas inhalation may approve this hypothesis (See Fig 3).

An early response to inhaled toxins, organic material, and inorganic material is the recruitment of macrophages to the lung, where they undergo phagocytosis and, if possible, destroy unwanted irritants. Macrophages in BAL fluid from the lungs of smokers and those patients with COPD are elevated many fold [49]. Furthermore, there is a positive association between macrophage numbers in the alveolar walls and the presence of mild-to-moderate emphysema as well as the degree of small airways disease in patients with COPD [49].

COPD is characterized by chronic obstruction of expiratory flow affecting peripheral airways, often associated with chronic bronchitis (mucus hypersecretion with goblet cell and submucosal gland hyperplasia) and emphysema (destruction of airway parenchyma). Tissue damage with airway wall remodeling and thickening, inflammation and fibrosis of the small airways appear to play an important role in patients with COPD. Increased numbers of neutrophils and macrophages are usually recovered in bronchoalveolar lavage fluid and induced sputum from such patients, and in the small airways, there is a mucosal increase in the numbers of CD8 T-cells. Alveolar macrophages play a significant and important role in inflammation in chronic obstructive pulmonary disease. The numbers of alveolar macrophages are markedly increased in the lungs of patients with COPD as a result of increased recruitment, proliferation and survival. In COPD, alveolar macrophages produce many inflammatory mediators, oxidants, proteins and proteinases in response to cigarette smoke extract and other stimuli. Macrophage numbers in the airways correlate with the severity of COPD [50-52]. In our study, a negative correlation between the FEV_1 _(% predicted) and both the percentage and the absolute number of macrophages may support the concept that the role of the macrophage in sulfur mustard gas-induced bronchiectasis may be marginal for the pathologic consequences in our veterans with bronchiectasis. On the other hand, our results showed that neutrophil numbers and percentages are directly related to the % FEV_1 _(% predicted). Therefore, it may be suggested that the neutrophil is a major player in our veterans who developed bronchiectasis.

The mainstay in pulmonary therapy of these patients bases on physiotherapy (bronchial clearing) inhalations [application of a variety of medications (bronchodilators, antibiotics, steroids)], and enteral or parenteral antibiotics to fight against bronchial infections. Macrolide treatment is now considered to be effective for diffuse panbronchiolitis (DPB), cystic fibrosis primarily through the inhibition of neutrophil accumulation in the lung [53-55]. The results of studies show that low- dose macrolide may improve respiratory symptoms in sulfur mustard injured patients too [56,57]. Therefore, the use of azithromycin or erythromycin clarithromycin on a long-term basis for infection control and inflammation modulation is suggested.

In conclusion, CD4 T cells in BAL fluid were significantly elevated in patients with bronchiectasis. Patients with higher grades of bronchial destruction expressed as HRCT scores, revealed higher percentages and the absolute number of CD4 T cells and a higher CD4/CD8 ratio. This, coupled with high levels of inflammatory mediators suggests that sulfur mustard gas-induced bronchiectasis is an ongoing inflammation. Our study also showed that, even in the absence of an exacerbation, there is a marked increase in the levels of cytokine in the BAL fluid.
